# Longitudinal Frequencies of Blood Leukocyte Subpopulations Differ between NOD and NOR Mice but Do Not Predict Diabetes in NOD Mice

**DOI:** 10.1155/2016/4208156

**Published:** 2016-02-04

**Authors:** Tanja Telieps, Meike Köhler, Irina Treise, Katharina Foertsch, Thure Adler, Dirk H. Busch, Martin Hrabě de Angelis, Admar Verschoor, Kerstin Adler, Ezio Bonifacio, Anette-Gabriele Ziegler

**Affiliations:** ^1^Institute for Diabetes and Obesity, Helmholtz Zentrum München, Ingolstädter Landstrasse 1, 85764 Neuherberg, Germany; ^2^Institute of Diabetes Research, Helmholtz Zentrum München and Forschergruppe Diabetes, Klinikum rechts der Isar, Technische Universität München, Ingolstädter Landstrasse 1, 85764 Neuherberg, Germany; ^3^German Mouse Clinic, Institute of Experimental Genetics, Helmholtz Zentrum München, Ingolstädter Landstrasse 1, 85764 Neuherberg, Germany; ^4^Institute for Medical Microbiology, Immunology and Hygiene, Technical University of Munich, Trogerstraße 30, 81675 Munich, Germany; ^5^German Center for Diabetes Research (DZD), Ingolstädter Landstrasse 1, 85764 Neuherberg, Germany; ^6^Forschergruppe Diabetes e.V., Ingolstädter Landstrasse 1, 85764 Neuherberg, Germany; ^7^School of Life Science Weihenstephan, Technical University of Munich, Alte Akademie 8, 85354 Freising, Germany; ^8^Institute for Systemic Inflammation Research, University of Lübeck, Ratzeburger Allee 160, 23538 Lübeck, Germany; ^9^DFG Research Center for Regenerative Therapies Dresden, Medical Faculty, Technische Universität Dresden, Fetscherstrasse 105, 01307 Dresden, Germany

## Abstract

Immune phenotyping provides insight into disease pathogenesis and prognostic markers. Trajectories from age of 4 to 36 weeks were modeled for insulin autoantibodies and for leukocyte subpopulations in peripheral blood from female NOD (*n* = 58) and NOR (*n* = 22) mice. NOD mice had higher trajectories of insulin autoantibodies, CD4^+^ and CD8^+^ T lymphocytes, B lymphocytes, IgD^+^IgM^−^ B lymphocytes, and NK cells and lower trajectories of CD4^+^CD25^+^ T lymphocytes, IgM^+^ B lymphocytes, granulocytes, and monocytes than NOR mice (all *p* < 0.001). Of these, only the increased IAA trajectory was observed in NOD mice that developed diabetes as compared to NOD mice that remained diabetes-free. Therefore, the profound differences in peripheral blood leukocyte proportions observed between the diabetes-prone NOD mice and the diabetes-resistant mice do not explain the variation in diabetes development within NOD mice and do not provide markers for diabetes prediction in this model.

## 1. Introduction

Type 1 diabetes is an autoimmune disease resulting from destruction of pancreatic ß-cells by infiltrating immune cells. The NOD mouse is an important model of spontaneous autoimmune diabetes that has been used to understand type 1 diabetes pathogenesis and develop therapeutics to prevent ß-cell destruction [1]. Similar to humans who have genetic susceptibility to type 1 diabetes and islet autoimmunity, not all NOD mice progress to overt diabetes, and progression to diabetes occurs at a variable time point after the start of autoimmunity [2, 3]. NOD mice have alterations in leukocyte populations as compared to other mouse strains [4, 5]. It is therefore possible that the extent of change may associate with the likelihood and rate of diabetes development in individual mice and that this may reveal markers that may help stratify the rate of progression to diabetes in man. We, therefore, quantified leukocyte populations in peripheral blood from NOD and NOR mice in biweekly intervals from 4 weeks to 36 weeks of age or until diabetes onset. We compared trajectories of T cell and B cell subpopulations and of NK cells, granulocytes, and monocytes between NOD and NOR mice and within NOD mice that progressed and did not progress to diabetes.

## 2. Material and Methods

### 2.1. Mice

NOD (*n* = 71) and NOR (*n* = 40) mice were longitudinally followed up (Suppl. Figure A.1 in Supplementary Material available online at http://dx.doi.org/10.1155/2016/4208156). In the experimental groups (NOD, *n* = 58 mice; NOR, *n* = 22 mice), follow-up included biweekly venous blood sampling (heparin coated tubes) for FACS phenotyping, determination of insulin autoantibody (IAA) levels, and blood glucose concentration and for assessment of weight. In addition, a control group of 13 NOD and 18 NOR mice was naturally followed up for growth and diabetes development without blood sampling to assess whether frequent blood sampling would affect diabetes development. All mice were kept under the same specific pathogen-free (SPF) conditions at the Institute of Diabetes Research, Helmholtz Zentrum München. Diabetes development was monitored by weekly measurement of urine glucose (Diastix, Bayer, Leverkusen, Germany) and confirmed by a venous blood sugar level >13.9 mmol/L. Mouse breeding and experiments were carried out in accordance with German legal guidelines and were approved by the local animal welfare authority (license number TVA 70/07).

### 2.2. IAA Measurement and Flow Cytometry

IAA was measured by radiobinding assay as previously described [6, 7]. Whole blood was used to determine T, B, and other cell subpopulations [8]. Erythrocytes were lysed using ammonium chloride-mediated lysis. Cells were resuspended and incubated with Fc-blocking solution and subsequently with surface antibodies for 15 minutes on ice (Suppl. Table A.1). Dead cells were excluded using propidium iodide-staining and leukocytes were gated via CD45. Data were acquired with an LSR II-HTS Cytometer (Franklin Lakes, NJ, USA). The resulting data were analyzed with FlowJo (FlowJo Enterprise, Oregon, USA) using Boolean gating (Suppl. Figure A.2).

### 2.3. Statistical Analysis

Kaplan-Meier survival analyses and log-rank statistics were used to estimate diabetes progression in NOD mice. Longitudinal trajectories of IAA, T, B, and other cell subpopulations were modeled using first-, second-, and third-order polynomial growth models for each measure [9] and model selection performed according to the Akaike information criterion [10]. Trajectories were compared between (a) NOD and NOR mice, (b) NOD mice that developed overt diabetes and NOD mice that did not develop diabetes and between NOD mice that progressed rapidly (by age of 21 weeks which was the median age of diabetes development in NOD mice that developed diabetes) and nondiabetic NOD mice. For comparisons, trajectories were analyzed until age of 30 weeks except for comparisons with the NOD mice that developed diabetes rapidly, which were analyzed until age of 21 weeks. The overall level of significance was set to a two-tailed *p* value of 0.05. For the group comparisons of the 14 cell subpopulations we accounted for multiple testing resulting in the Bonferroni corrected significance level of 0.004. Longitudinal trajectories were visualized by time pointwise means and confidence intervals (CI) and smoothed locally via LOESS [11].

## 3. Results and Discussion

### 3.1. Results

**Figure 1:**
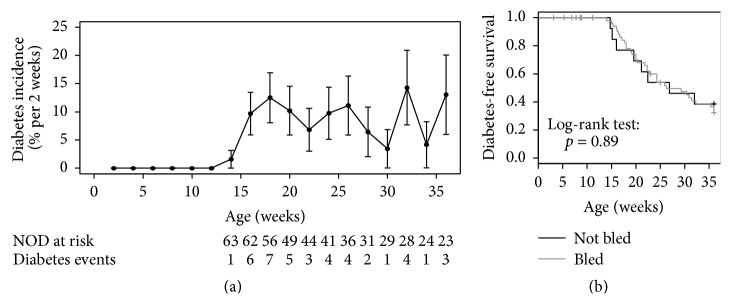
Diabetes development shown (a) as an incidence in all NOD mice followed up in the study and (b) as diabetes-free survival comparing NOD mice that were bled biweekly (black line) and NOD mice that were followed up without blood draws (gray line; *p* = 0.89).

In the experimental groups, 33 of the 58 female NOD mice developed diabetes at a median age of 22 weeks (IQR 18 to 27 weeks) and a cumulative risk at age of 36 (end of the study) of 32% (CI 21%–49%). None of the 22 NOR mice developed diabetes during follow-up. The incidence of diabetes in NOD mice was stable from age of 16 weeks (9% per 2 follow-up weeks from age of 10 weeks to age of 30 weeks, Figure 1(a)). No difference in diabetes development was observed between the NOD mice that were bled weekly as part of the study (57% by age of 36 weeks) and NOD mice that were not bled (62%, *p* = 0.89), indicating that frequent blood sampling did not affect diabetes development (Figure 1(b)). Weights were comparable between the NOR and NOR mice and between the NOD mice that developed diabetes and NOD mice that remained diabetes-free (Suppl. Figure A.3).

#### 3.1.1. IAA Titers Predicted Disease Progression in NOD Mice

**Figure 2:**
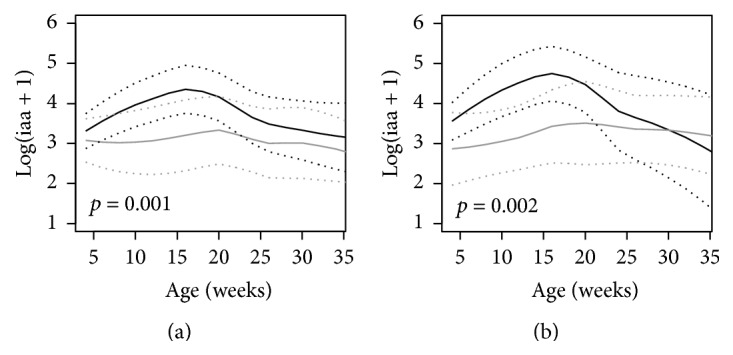
Trajectories of IAA in (a) NOD mice (black line) versus NOR mice (gray line) and (b) NOD mice that developed diabetes (black line) versus NOD mice that were diabetes-free at age of 36 weeks (gray line). The unbroken lines represent 95% confidence intervals of the trajectories.

Trajectories of IAA were higher in NOD mice than in NOR mice (Figure 2(a), *p* = 0.001) and higher in NOD mice that developed diabetes as compared to NOD mice that did not develop diabetes (Figure 2(b), *p* = 0.002).

#### 3.1.2. Leukocyte Populations in NOD and NOR Mice

**Figure 3:**
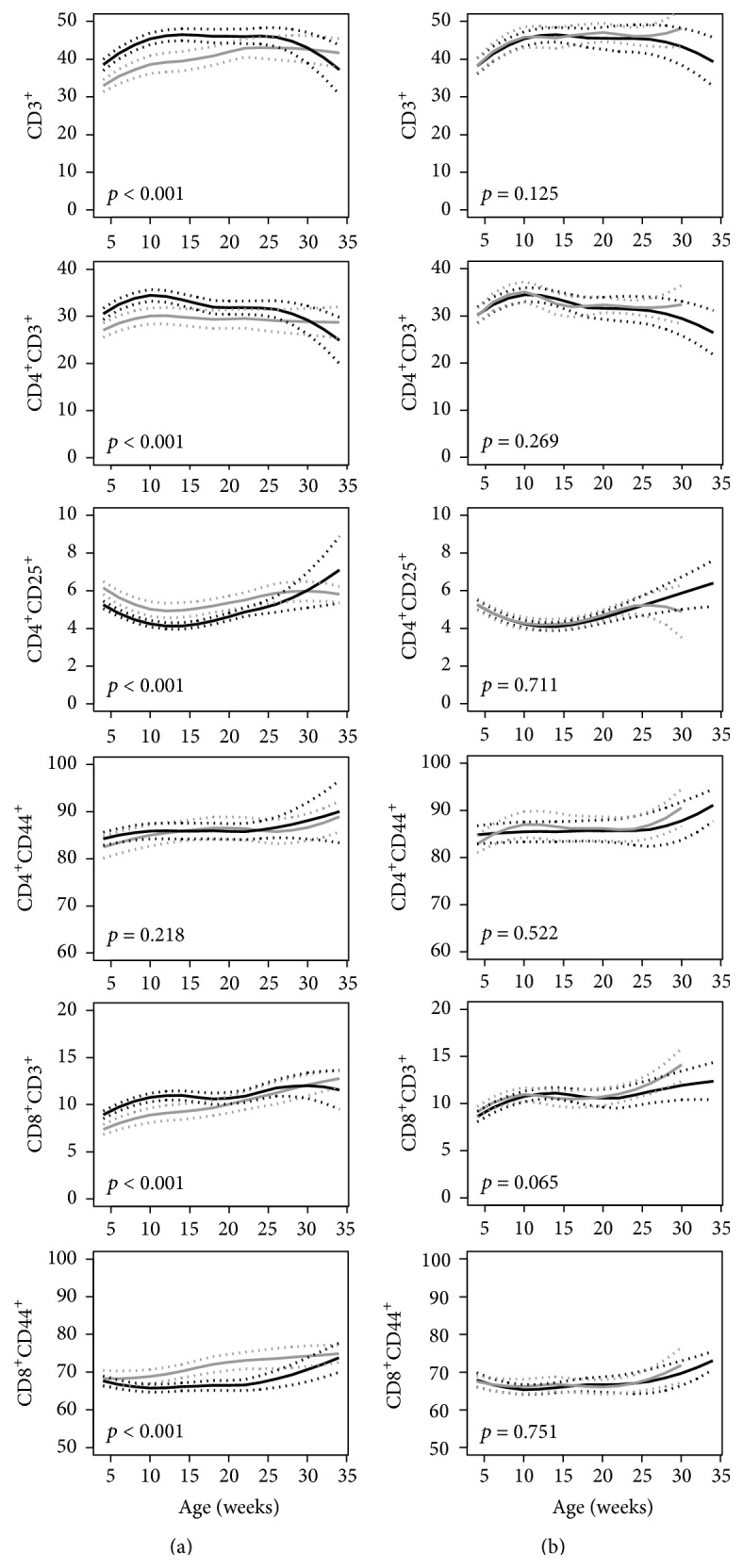
Trajectories of peripheral blood percentages of T lymphocyte populations in (a) NOD mice (black line) versus NOR mice (gray line) and (b) NOD mice that developed diabetes (black line) versus NOD mice that were diabetes-free at age of 36 weeks (gray line). The unbroken lines represent 95% confidence intervals of the trajectories. *p* values for the comparisons are shown in each graph.

*T Cells.* Trajectories of total T cells (CD3^+^), T helper cells (CD4^+^CD3^+^), and cytotoxic T cells (CD8^+^CD3^+^) were higher in NOD mice compared to NOR mice (*p* < 0.001, Figure 3(a)). Despite this, CD4^+^CD25^+^ regulatory T cells and the CD44^+^ subpopulation of CD8^+^ T cells were lower in NOD mice compared to NOR mice (*p* < 0.001, Figure 3). No differences were found for any of the T cell populations between NOD mice progressing to diabetes and NOD mice that remained diabetes-free (Figure 3(b)), nor between the NOD mice that developed diabetes early as compared to mice that remained diabetes-free (Suppl. Figure A.4).

**Figure 4:**
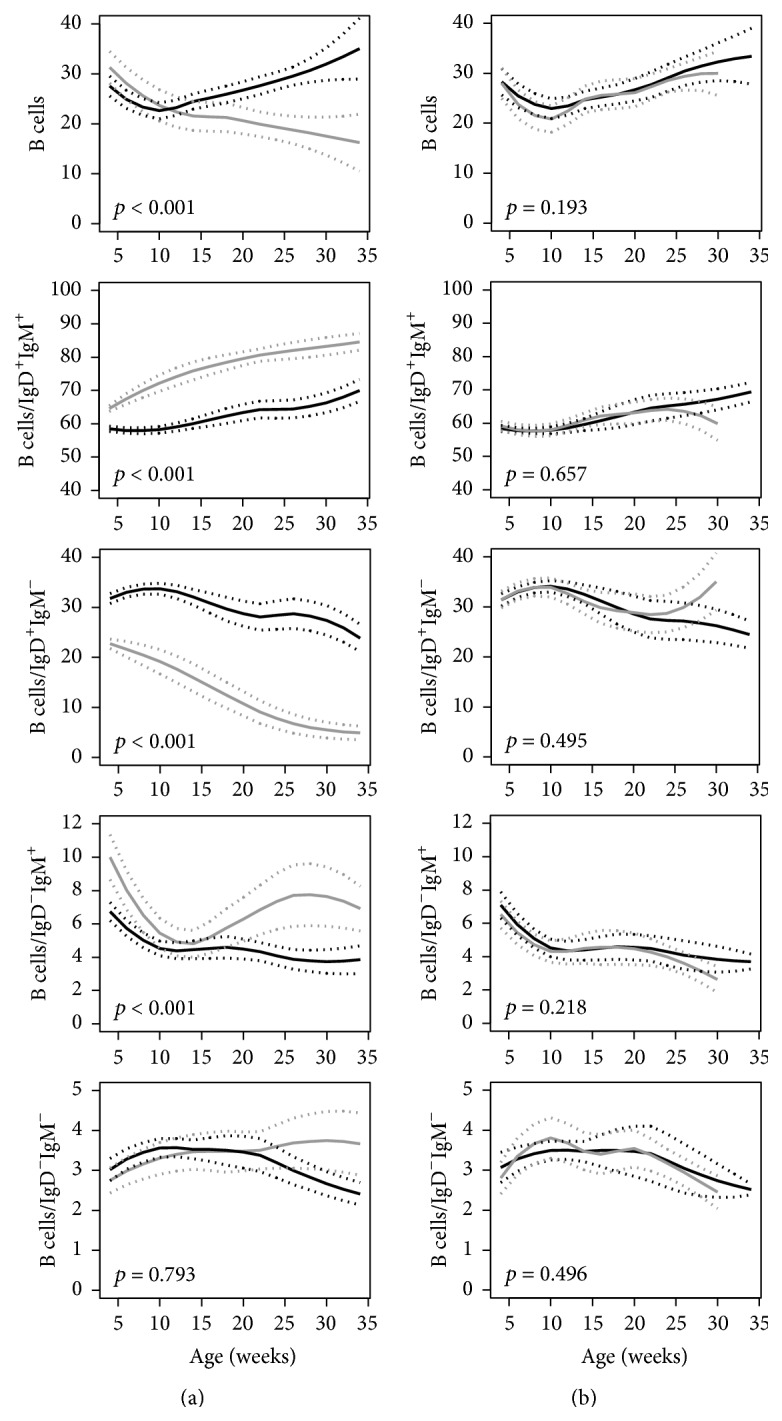
Trajectories of peripheral blood percentages of B lymphocyte populations in (a) NOD mice (black line) versus NOR mice (gray line) and (b) NOD mice that developed diabetes (black line) versus NOD mice that were diabetes-free at age of 36 weeks (gray line). The unbroken lines represent 95% confidence intervals of the trajectories. *p* values for the comparisons are shown in each graph.

*B Cells.* Trajectories of total B cell frequencies (CD19^+^ and CD19^low^B220^+^IgD^+^) and B cell subpopulations were significantly different between NOD and NOR mice with NOD mice presenting higher frequencies of later stage B cells (*p* < 0.001, Figure 4). Within the B cell compartment, NOR mice showed a higher percentage of naïve (IgD^+^IgM^+^) and activated (IgD^−^IgM^+^) B cells, whilst NOD mice had higher percentages of IgD^+^ IgM^−^ cells (Figure 4). The trajectories of B cells and B cell subpopulations were similar between NOD mice that progressed to diabetes and NOD mice that remained diabetes-free (Figure 4).

**Figure 5:**
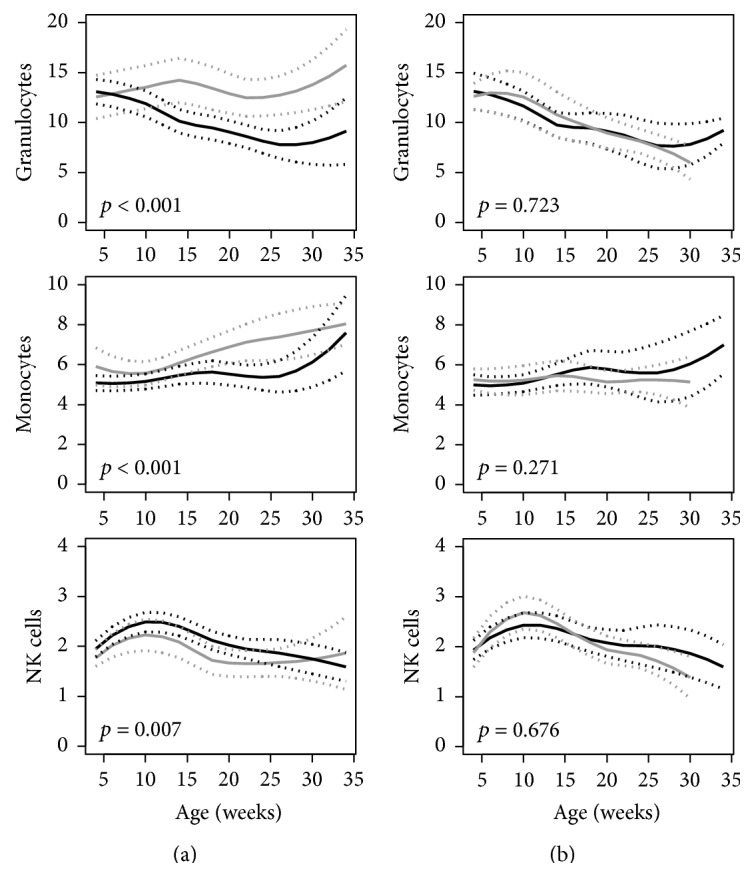
Trajectories of peripheral blood percentages of granulocytes, monocytes, and NK cells in (a) NOD mice (black line) versus NOR mice (gray line) and (b) NOD mice that developed diabetes (black line) versus NOD mice that were diabetes-free at age of 36 weeks (gray line). The unbroken lines represent 95% confidence intervals of the trajectories. *p* values for the comparisons are shown in each graph.

*Other Leukocytes.* The trajectories of granulocyte and monocyte frequencies were lower in NOD mice compared to NOR mice and the trajectory of NK cell frequencies was higher in the NOD mice (Figure 5). No differences were found within NOD mice relative to diabetes progression (Figure 5).

### 3.2. Discussion

Autoimmunity likely results from the summation of multiple altered tolerance and immune response mechanisms. Here we used the NOD mouse model to portray longitudinal alterations of multiple leukocyte populations from birth to age of 36 weeks with the intention to identify potential biomarkers that may associate with the likelihood and rate of autoimmune diabetes development. We found profound differences in the circulating B cell compartment of NOD compared to NOR and additionally significant but less pronounced differences in T cells, granulocytes, and monocytes. However, none of these differences were able to predict which NOD mice would develop diabetes or when the mice would develop diabetes. The only assessed immune marker which distinguished progressor from nonprogressor NOD mice was the autoantibody response to insulin as previously described [12].

The study is unique because it included prospective frequent phenotyping and utilized advanced statistical modeling of longitudinal data for the analysis. Additionally, the study utilized peripheral blood and therefore mimicked sample availability in humans. Limitations include the low blood volume that restricts how many cell populations were examined as well as the ability to precisely measure rare cell phenotypes. Since we did not examine cells in lymphoid organs and in the pancreas, we do not know if any of the observed alterations are generalized to the immune and hematopoietic systems.

Some of the findings in the NOD mice were similar to what is observed in man. The relationship between increased IAA and more diabetes development is reflected by the faster rate of progression in antibody children if they have an earlier seroconversion or have higher islet autoantibody titers [13, 14]. Similar to NOD mice, although much less pronounced, girls with islet autoantibodies progress to diabetes faster than boys [13]. Finally, it appears that both NOD mice and multiple islet autoantibody positive children progress to diabetes at a constant rate. In mice, this is from around 10 weeks of age in our colony, while in children it is from the time of seroconversion [15]. Such a constant rate raises the possibility that progression is stochastic rather than induced by single events.

NOD mice are reported to have alterations in immune cell numbers and function [16, 17]. We confirm this and show that, as compared to genetically similar NOR mice, they have more circulating CD4^+^ and CD8^+^ T cells but lower circulating CD4^+^CD25^+^ T cells. Most striking were the novel differences observed in the B cell population with a more general evidence of B cell maturation from early age in the NOD mice. Fiorina and colleagues describe that depletion of mature (CD22-positive) B cells reverses autoimmune diabetes in NOD mice, which argues for a role of B cells in diabetes development in NOD mice [18]. More subtle differences in circulating B cell subpopulations have also been observed in patients with type 1 diabetes [19]. We confirmed that NOR mice have insulin autoantibodies, but to a lower extent than NOD mice as previously demonstrated [20]. Circulating monocyte and granulocyte frequencies also differed in the NOD mice indicating more general hematopoietic changes. Some of the observed differences in blood cell populations may provide susceptibility to developing autoimmunity. Disappointingly, however, none of the differences were in any way related to whether the NOD mice progressed to diabetes, suggesting that they neither contribute to progression nor reflect other changes that occur during progression.

## 4. Conclusions

Longitudinal trajectories of leukocyte subpopulations were not found to be valuable biomarkers to predict disease progression in NOD mice.

## Supplementary Material

Figure A.1: Overview of the study design.
Figure A.2: FACS Gating strategy for leukocytes, T cells, Granulocytes, Monocytes, NK and B cells.
Figure A.3: Weight during the follow up of NOD and NOR mice as well as NOD mice with and without diabetes progression.
Figure A.4: Trajectories for additional cell populations in NOD and NOR mice.
Table A.1: Cell populations analyzed via FACS and Boolean Gating.

## Abbreviations

CI: Confidence interval
IQR: Interquartile Range
SPF: Specific pathogen-free
TVA: Tierversuchsantrag.
