# Supplementary material for: Longitudinal Frequencies of Blood Leukocyte Subpopulations Differ between NOD and NOR Mice but Do Not Predict Diabetes in NOD Mice
Source: J Diabetes Res. 2016 Feb 4;2016:4208156. doi: 10.1155/2016/4208156 (PMC4757706; doi:10.1155/2016/4208156)
Supplement: Supplementary file 1 — Figure A.1: Overview of the study design. Figure A.2: FACS Gating strategy for leukocytes, T cells, Granulocytes, Monocytes, NK and B cells. Figure A.3: Weight during the follow up of NOD and NOR mice as well as NOD mice with and without diabetes progression. Figure A.4: Trajectories for additional cell populations in NOD and NOR mice. Table A.1: Cell populations analyzed via FACS and Boolean Gating. [file 4208156.f1.docx]

# Supplemental Figures

**
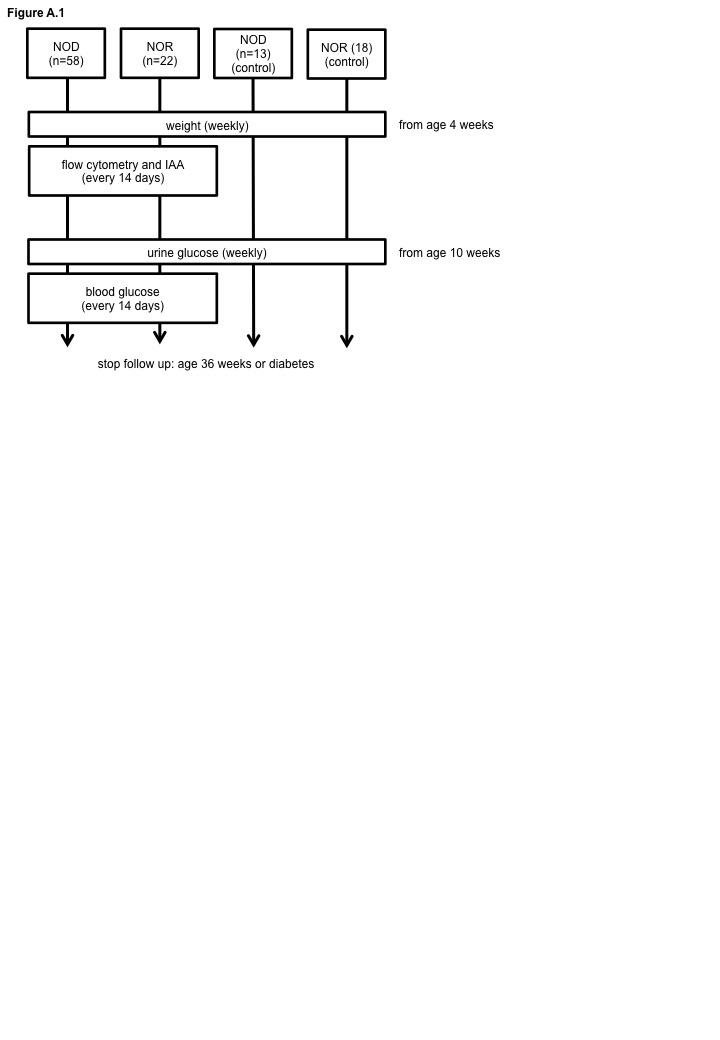
**

Study design


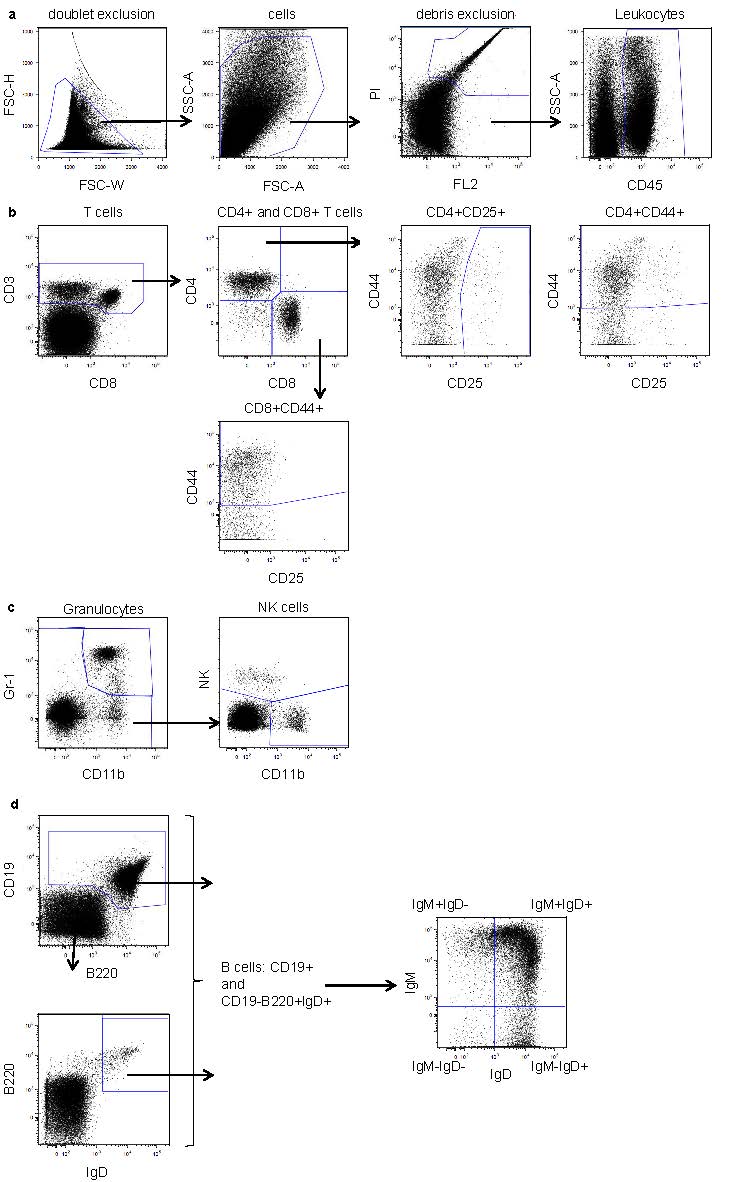
**Figure A.2**

FACS Gating strategy for (a) Leukocytes (used for all stainings) (b) T cells (c) Granulocytes, Monocytes and NK cells (d) B cells (CD19+ and CD19-B220+ cells

were gated as B cells); Antibodies used: CD44, IgD, CD4, Gr1, CD138, CD19, CD25,

CD5 (BD Bioscience), GARP, NKp46, CD62L, CD3 (eBioscience), CD11b, CD8a, B220

(Life Tchnologies) and CD45 (BioLegend)

**
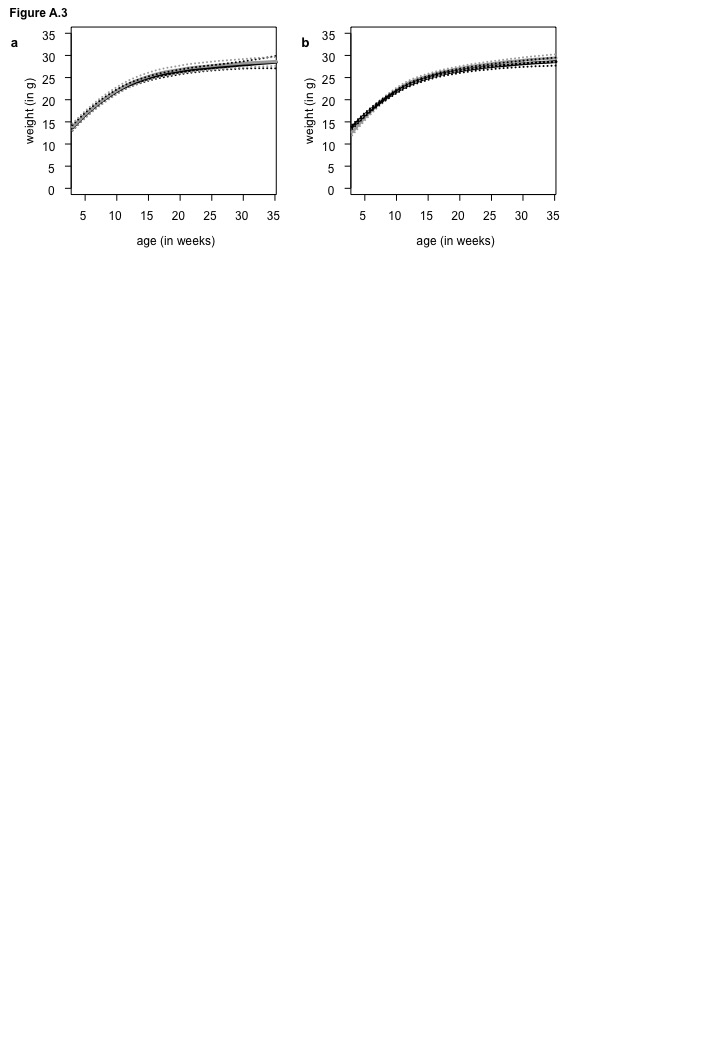
**

Weight in (a) NOD mice (black line) vs NOR mice (gray line), and (b) NOD

mice that developed diabetes (black line) vs NOD mice that were diabetes-free at age

36 weeks (gray line). The unbroken lines represent 95% confidence intervals.


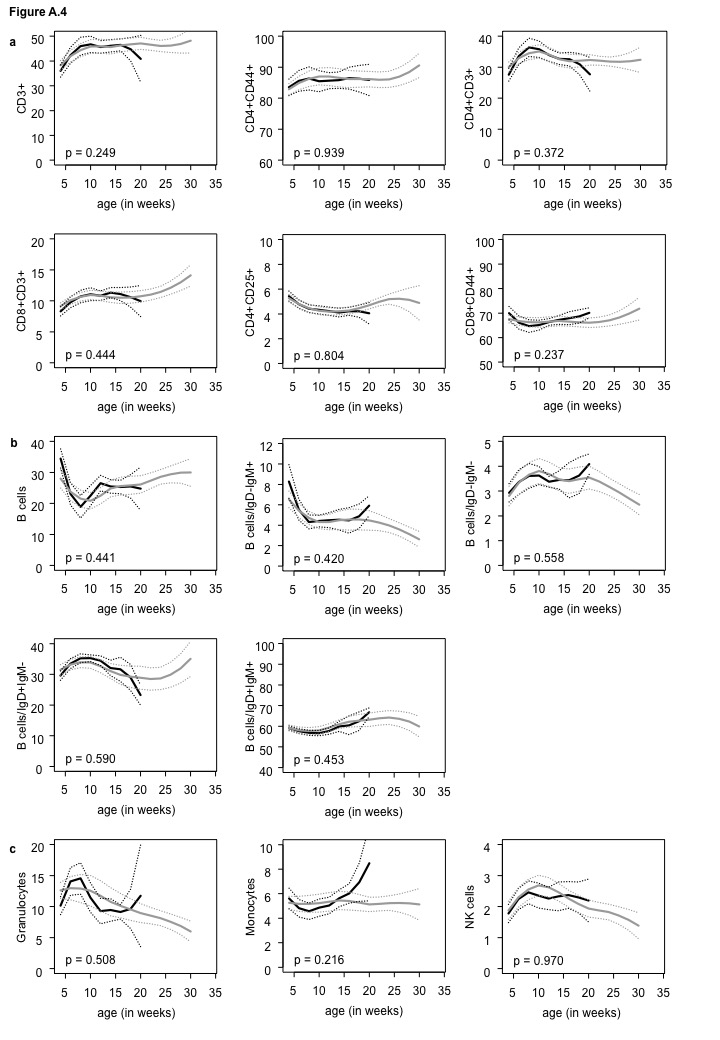


Trajectories of peripheral blood percentages of T lymphocyte, B lymphocyte

populations, granulocyte, monocyte, and NK cells in NOD mice that developed diabetes

by age 21 weeks (black line) vs NOD mice that were diabetes-free at age 36 weeks (gray line). The unbroken lines represent 95% confidence intervals of the trajectories. P

values for the comparisons are shown in each graph.

# 8. Tables (Supplement)

**Table . A.1 Cell populations analyzed via FACS and Boolean Gating**

**Marker Name parental population**

**T cells**

CD3^+^ T cells % of leukocytes

CD8^+^CD3^+^ Cytotoxic T cells % of leukocytes

CD4^+^CD3^+^ T helper cells % of leukocytes

CD4^+^/CD25^+^ Tregs (CD25^+^ T cells) % of CD4 T cells

CD4^+^/CD44^+^ CD44^+^ T helper cells % of CD4 T cells

CD8^+^/CD44^+^ CD44^+^ cytotoxic T cells % of CD8 cells

**Granulocytes, Monocytes, NK cells and B cells**

Granulocytes Granulocytes % of leukocytes

Monos Monocytes % of leukocytes

CD45^+^/NK^+^ NK cells % of leukocytes

CD19^+^ CD19^+^ B cells % of leukocytes

B cells CD19^+^& CD19^low^B220^+^IgD^+^B cells % of leukocytes

B cells/CD19^+^ CD19^+^ B cells % of B cells

B cells /IgD^+^ IgD^+^ B cells % of B cells

B cells /IgM^+^ IgM^+^ B cells % of B cells

B cells /CD11b^+^ CD11b^+^ B cells % of B cells

B cells /IgD^+^IgM^+^ peripheral B cells % of B cells

B cells /IgD^+^IgM^-^ IgD^-^only B cells % of B cells

B cells /IgD^-^IgM^+^ peripheral activated B cells % of B cells

B cells /IgD^-^IgM^-^ IgD^-^IgM^-^ B cells % of B cells

**B cells - only**

B cells B cells % of leukocytes

B cells /CD5^+^ B1 cells/CD5^+^ B cells % of B cells

B cells /IgD^+^ IgD^+^ B cells % of B cells

B cells /CD11b^+^  CD11b^+^ B cells % of B cells
